# Circulating Actin-Binding Proteins in Laryngeal Cancer: Its Relationship with Circulating Tumor Cells and Cells of the Immune System

**DOI:** 10.32607/actanaturae.11413

**Published:** 2021

**Authors:** G. V. Kakurina, M. N. Stakheeva, I. A. Bakhronov, E. E. Sereda, O. V. Cheremisina, E. L. Choynzonov, I. V. Kondakova

**Affiliations:** Cancer Research Institute, Tomsk National Research Medical Center, Russian Academy of Sciences, Tomsk, 634050 Russia

**Keywords:** actin-binding proteins, circulating tumor cells, leukocytes, laryngeal squamous cell carcinoma

## Abstract

We previously exposed the role of actin-binding proteins (ABPs) in cancer development and progression. In this paper, we studied the relationship between circulating ABPs and the number of ABP-expressing leukocytes and circulating tumor cells (CTCs) in patients with highly aggressive laryngeal squamous cell carcinoma (LSCC). The levels of cofilin (CFL1), profilin (PFN1), ezrin (EZR), fascin (FSCN1), and adenylate cyclase-associated protein 1 (CAP1) were determined using enzyme immunoassay. The ABP expression by the cellular pools was analyzed by flow cytometry. The highest levels of FSCN1 and EZR were found in the blood serum of LSCC patients. There was a difference in ABP expression between the pools of leukocytes and CTCs. Leukocytes were mainly represented by CAP1+ and FSCN1+ pools, and CTCs contained CAP1+, FSCN1+, and EZR+ cells. The serum FSCN1 level correlated with the number of FSCN1-containing and CFL1-containing leukocytes. Thus, the level of circulating EZR is likely related to its expression in CTCs. The levels of CFL1 and PFN1 are likely to be supported by the expression of these proteins by leukocytes. Both CTCs and leukocytes can be a source of FSCN1 and CAP1 in blood serum. The results suggest that serum proteins can be produced by various cells, thus indicating both cancer development and the response of the immune system to this process.

## INTRODUCTION

Metastases are considered to be the major cause of death in cancer patients. It is important to study metastasis-related biological processes in order to attempt to identify prognostic markers of tumor progression [1]. Research focusing on blood serum/plasma proteome profiling of cancer patients using mass spectrometry is ongoing. Laryngeal squamous cell cancer (LSCC) is one of the aggressive cancers, which makes it a good model for studying the metastasis mechanisms [2, 3, 4]. Earlier, we uncovered the differences in the blood serum proteome of LSCC patients and healthy volunteers, as well as the correlation between several functionally different proteins (including actin-binding protein CAP1 (adenylyl cyclase-associated protein 1)) [4] and metastases of LSCC. Actin-binding proteins (ABPs) coordinate the rearrangement of the actin cytoskeleton, which is closely linked to metastasis development. The ABP level in tumors has been investigated rather thoroughly [5, 6, 7], while systemic circulating ABPs (cABPs) have been insufficiently studied. Previously, the serum levels of CAP1, profilin 1, and fascin 1 in T3-4N0–1M0 LSCC patients were found to differ from those in patients with T1N0M0 LSCC [8]. It is possible that the cABP level in systemic circulation can be maintained by several sources, including immune circulating tumor cells (CTCs). The correlation between cABPs and their potential cellular sources in systemic circulation is virtually unstudied. Therefore, in this work, the serum level of cABP was compared to cABP expression in populations of leukocytes and CTCs in systemic circulation in order to identify any relationship between these parameters. Peripheral blood samples from LSCC patients were used as a model of aggressive cancer with a high probability of metastasis.

## EXPERIMENTAL

**Material and characterization of patient groups**

The study involved 13 LSCC patients (stage T2-4N0-2M0) (four patients, T2-4N0M0; nine patients, T2-4N1-2M0) with a morphologically verified diagnosis, who were not receiving antitumor therapy. The patients’ mean age was 57 (52–63) years. Blood serum for ELISA was collected in accordance with an approved protocol and stored at -80°C. Freshly collected blood samples were used for flow cytometry. All the manipulations were conducted after the patients had provided informed consent, and patients’ confidentiality was maintained in compliance with the World Medical Association’s Declaration of Helsinki "Ethical Principles for Medical Research Involving Human Subjects" (as amended in 2000). The study was approved by the Ethics Committee of the Cancer Research Institute, Tomsk National Research Medical Center. All study subjects had signed informed consent forms.

**Study methods**

The analysis of cABPs in peripheral blood was performed by enzyme-linked immunosorbent assay on a Multiscan FC microplate reader (Thermo Fisher Scientific, USA) according to the instructions in the kit. The following ELISA kits (Cloud-Clone Corp.) were used: CAP1 (SEB349Hu), PFN1 (SEC233Hu), CFL1 (SEB559Hu), FSCN1 (EB757Hu), and EZR (SEB297Hu).

ABP expression in leukocytes and CTCs was analyzed by flow cytometry on a BD FACS Canto II cytometer (BD, USA). The total leukocyte pool and CTC populations were identified using blood cell labeling with the specific fluorescent tags CD45 (AF700 (BD)) and EpCAM (PerCPCy5.5 (BD)), respectively. ABP expression in the cell pools was assessed using AF488- conjugated mouse monoclonal antibodies against human ezrin (pY353) (BD); APC-conjugated rabbit polyclonal antibodies against human CFL1 (Cloud- Clone Corp); AF647-conjugated rabbit polyclonal antibodies against human PFL1 (Cloud-Clone Corp); PE-conjugated rabbit polyclonal antibodies against human FSCN1 (Biorbyt); unconjugated rabbit monoclonal antibodies against human CAP1 (Abcam, UK); and goat anti-rabbit antibodies conjugated to AF488 (Abcam) as secondary anti-CAP1 antibodies. The gating strategy involved the separation of blood cells into CD45+ cells (leukocytes) and CD45- cells. Gates of CAP1+, EZR+, PFL1+, CFL1+, and FSCN1+ cells were isolated from the gate of CD45+ leukocytes, and their percentage in the total leukocyte pool was determined. CD326 (EpCAM)+ cells were isolated from the gate of CD45- cells by regarding them as circulating tumor cells; the aforelisted populations were sequentially isolated from CTCs CD326 (EpCAM)+, and their percentage in the pool of CTCs was assessed. The results were presented as a percentage of CD45+ and EpCAM+ cells expressing these ABPs.

**Statistical analysis**

The data were analyzed using the IBM SPSS Statistics 22.0 software. The existence of correlation and its strength were evaluated using the Spearman’s rank correlation coefficient (r). The results were presented as Me (Q1; Q3), where Me is the median value; Q1 and Q3 are the upper and lower quartiles, respectively. Differences at p ≤ 0.05 were considered statistically significant.

## RESULTS AND DISCUSSION

**Fig. 1:**
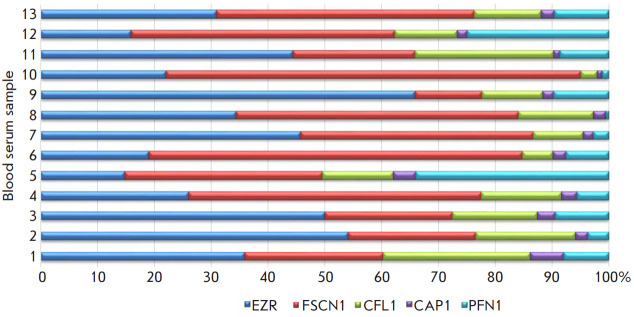
The serum levels of circulating actin-binding proteins in LSCC patients. The Y axis shows patients with LSCC, and the X axis shows the percentage concentration of serum ABPs, % of the total cABP content (assumed to be 100%)

We determined the serum levels of cABP in LSCC patients by ELISA. The FSCN1 and EZR levels were the highest: the median levels were 1.8 (0.43–8.1) and 2.1 (1.69–2.56) ng/mL, respectively. The CAP1 level was the lowest (median value = 0.11 (0.08–1.15)), followed by the levels of PFN1 and CFL1 (median values: 0.28 (0.23–0.38) and 0.78 (0.63–1.14) ng/mL, respectively). Figure 1 shows the dispersion of the serum levels of each protein in LSSC patients.

**Fig. 2:**
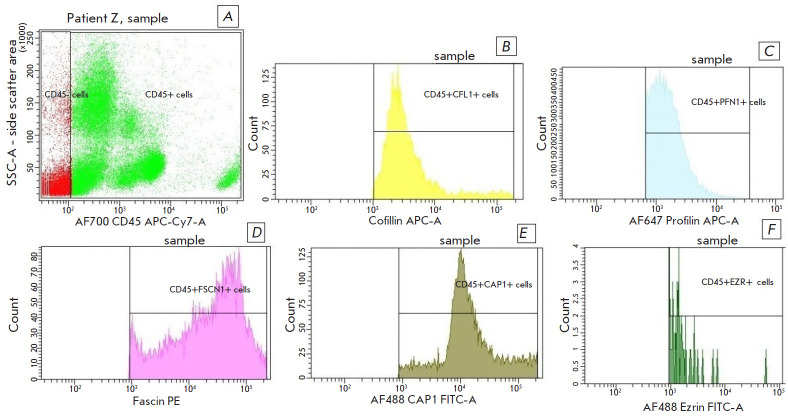
The relative number of CD45+ cells expressing actin-binding proteins in patients with laryngeal cancer. (*A*) – Gates of CD45- and CD45+ peripheral blood cells. Histograms representing the content of actin-binding proteins in CD45+ cells are shown in the figures: (*B*) – the count of CD45+ cells containing CFL1; (*C*) – the count of PFN1-containing CD45+ cells; (*D*) – the count of FSCN1-containing CD45+ cells; (*E*) – the count of CAP1-expressing and (*F*) – the count of EZR-containing CD45+ cells

**Fig. 3:**
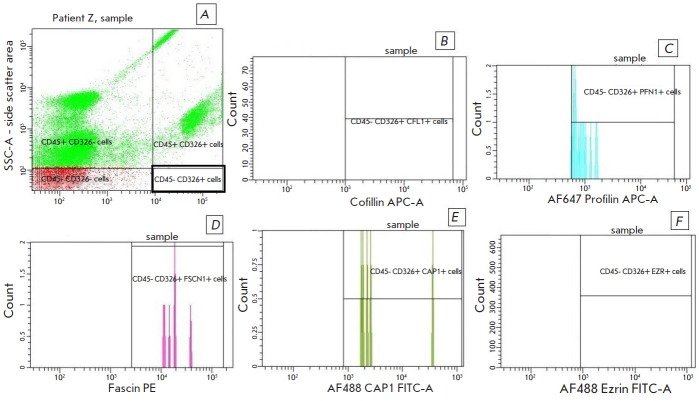
The relative number of circulating tumor cells (CD45-CD326+) containing actin-binding proteins in peripheral blood in patients with laryngeal cancer. (*A*) – Gate of CD45-CD326+ cells (CSCs) in peripheral blood. Histograms showing the contents of actin-binding proteins in CTCs are shown in the figures: (*B*) – the level of CTCs containing CFL1; (*C*) – the level of PFN1-containing CSCs; (*D*) – the level of fascin-containing CTCs; (*E*) – the level of CAP1-expressing; and (*F*) – the level of EZR-containing CTCs

The ABP levels in the pools of CTCs (CD45- CD326+) and leukocytes (CD45+CD326-) in the whole blood of LSCC patients were then determined. It was shown for the presented sample of LSCC patients that the median level of CTCs CD45-CD326+ was 0.006 (0.00–0.1) % of all cellular components of the blood (per 50,000 blood cells). Differences in the relative content of all ABPs in the populations of CD45- CD326+ CTCs and CD45+ leukocytes were revealed (Fig. 2, Fig. 3).

Table shows the relative number of CD45-CD326+ CTCs and CD45+ leukocytes expressing ABPs in LSCC patients. CD45-CD326+ CTCs in LSCC patients predominantly consist of FSCN1+ and CAP1+ subpopulations whose median percentage was 91.8 (87.2– 100) % and 87.0 (61.5–100) %, respectively. The percentage of CD45-CD326+ CTCs expressing PFN1 and CFL1 was reduced: 0.2 (0.0–0.5) % and 0.3 (0.0–0.5) %, respectively. The population of CD45+ leukocytes is mainly represented by CAP1+ and FSCN1+ cells: 45.3 (4.6–55.3) % and 34.5 (31.3–72.1) %, respectively. The percentage of the EZR+ CD45+ leukocyte subpopulation was reduced (0.3 (0.14–0.91) %). EZR was mainly expressed by CTCs (51.5 (39.3–85.4) %).

**Table 1:** The serum level of actin-binding proteins (ABPs), the relative number of CD45-CD326+ circulating tumor cells and CD45+ leukocytes expressing actin-binding proteins in patients with laryngeal squamous cell carcinoma

cABP	Leukocytes, CD45+, %	CTCs, CD45-CD326+, %	Blood serum, ng/mL
EZR	0.6 (0.3–1.0) 51.5	(39.3–85.4) 1.2	(0.9–1.7)
FSCN1	34.5 (31.3–72.1)	91.8 (87.2–100)	1.5 (0.8–2.2)
CFL1	12.0 (8.5–39.1)	0.3 (0.0–0.5)	0.5 (0.3–0.6)
CAP1	45.3 (4.6–55.3)	87.0 (61.5–100)	0.10 (0.06–0.14)
PFN1	5.7 (4.0–13.2)	0.2 (0.0–0.5)	0.2 (0.1–0.4)

An analysis of the correlations between the cABP level and the number of cell subpopulations expressing the respective protein revealed medium-strength correlations. Thus, the level of circulating FSCN1 correlated with the percentage of FSCN+ and CFL1+ subpopulations of CD45+ leukocytes (r = 0.7; p = 0.03). Correlations between the analyzed pools expressing ABPs were also revealed in the blood circulation of LSSC patients. The pool of CD45-CD326+ leukocytes containing FSCN+ was found to negatively correlate with CD45+ leukocytes containing FSCN+ (r= -0.7; p = 0.01) and CFL1+ (r = -0.7; p = 0.03). A positive correlation was revealed between CAP1+ CD326+ and CAP1+ CD45+ (r = 0.7; p = 0.02). A correlation between EZR+ and CFL1+ CD45+ leukocytes was revealed at the level of the leukocyte pool.

This study showed that the levels of the FSCN1 and EZR proteins in the systemic circulation of LSCC patients were higher compared to the levels of other cABPs. Peripheral blood leukocytes and CTCs were found to differ in ABP expression. A correlation between the serum level of FSCN1 and the percentage of FSCN+ and CFL1+ CD45+ leukocytes was revealed. CAP1 and FSCN1 expressions were increased in both cell populations. However, significant differences in the levels of these proteins in CTCs and leukocytes were detected. Whereas almost all CTCs contained CAP1 and FSCN1, the percentages of CAP1+ and FSCN1+ leukocytes were lower by two-and threefold, respectively. The population of CD45+ leukocytes had almost no EZR+ cells, while the percentage of EZR+ CD326+ was 51.5% of the pool of CTCs. CFL1 and PFN1 were found in leukocytes but flow is associated with its presence in CTCs. The level of CFL1 and PFN1 circulating in the blood is probably maintained by the expression of these proteins by leukocytes. The serum level of FSCN1 and CAP1 can be related both to CTCs and to leukocytes.

The involvement of ABPs in the pathogenesis of cancer has been, for the most part, studied based on the results of a determination of their tissue levels [7, 9, 10]. It has been suggested that the fascin levels in the tissues of head and neck tumors can be used as prognostic markers of tumors of this localization [https://www.proteinatlas.org; 11]. The results of the study of ABPs in neoplasm tissues (tumor cells being the most plausible and the main source of these proteins) have been reported. The role played by cABPs in patients with pathological conditions, including cancer, has been very poorly studied. For example, it has been shown that extracellular gelsolin (pGSN) cleaves actin, which can be released upon cell injury [12]. The ABPs studied in this publication also may be functionally active in blood serum, which can become the subject of further research.

## CONCLUSIONS

Data on a correlation between functionally different systemically circulating ABPs and populations of CTCs and immune cells expressing the respective proteins in patients with laryngeal cancer were obtained for the first time in this study. The revealed correlations and differences in the ABP level in CTCs and leukocytes can be attributed both to the specificity of a tumor of this localization and/or be indicative of the body’s overall immune response to tumor growth. To draw any definitive conclusions, the research needs to be continued, with a larger number of LSCC patients and an assessment of additional clinical and morphological parameters.

## Abbreviations

LSCC: laryngeal squamous cell carcinoma
ABPs: actin-binding proteins
CTCs: circulating tumor cells
CAP1: adenylyl cyclase-associated protein 1
CFL1: cofilin
PFN1: profilin 1
EZR: ezrin
FSCN1: fascin
